# Association Between Chronic Tamsulosin Therapy and Intraoperative Hemodynamics During Spinal Anesthesia: A Prospective Observational Study

**DOI:** 10.3390/jcm15207978

**Published:** 2026-10-15

**Authors:** Tuba Kuvvet Yoldaş, Nevin Esra Gümüş, Aylin Ateşalp, Mehmet Yoldaş

**Affiliations:** 1 Department of Anesthesiology and Reanimation, İzmir City Hospital, İzmir 35530, Türkiye; 2 Department of Anesthesiology and Reanimation, Samsun City Hospital, Samsun 55080, Türkiye; 3 Department of Urology, İzmir Tepecik Training and Research Hospital, University of Health Sciences, İzmir 35180, Türkiye

**Keywords:** Tamsulosin, spinal anesthesia, Hypotension, hemodynamics, transurethral resection of the prostate, benign prostatic hyperplasia

## Abstract

**Background**: This prospective observational study investigated the association between chronic tamsulosin therapy and intraoperative hemodynamic changes in patients undergoing elective transurethral resection of the prostate (TUR-P) under spinal anesthesia. **Methods**: Adult patients scheduled for elective TUR-P under spinal anesthesia were prospectively enrolled. Patients receiving long-term tamsulosin therapy comprised the tamsulosin group, whereas those with no history of α1-adrenergic blocker use served as the control group. Hemodynamic variables, including mean arterial pressure (MAP) and heart rate, were recorded before spinal anesthesia and at predefined time points during the first 60 min after spinal anesthesia. **Results**: A total of 115 patients were analyzed (tamsulosin, *n* = 56; control, *n* = 59). Baseline MAP was comparable between groups. The adjusted linear mixed-effects model demonstrated a significant group × time interaction for MAP (F(5, 565.10) = 8.34, *p* < 0.001). Adjusted between-group MAP differences were −4.9 mmHg (95% CI, −8.8 to −1.0; *p* = 0.015) at 5 min, −17.0 mmHg (95% CI, −20.2 to −13.8; *p* < 0.001) at 30 min, and −16.4 mmHg (95% CI, −19.5 to −13.3; *p* < 0.001) at 60 min. Criteria-defined hypotension occurred in 21/56 (37.5%) patients in the tamsulosin group and 17/59 (28.8%) in the control group (*p* = 0.32). **Conclusions**: Chronic tamsulosin therapy was associated with lower intraoperative MAP values during spinal anesthesia, without a statistically significant increase in the incidence of clinically defined hypotension. These findings suggest that chronic tamsulosin use may be associated with a different intraoperative blood pressure response following spinal anesthesia.

## 1. Introduction

Alpha-1 adrenergic receptor antagonists are widely used as first-line pharmacological therapy for men with moderate-to-severe lower urinary tract symptoms, as recommended by current European Association of Urology guidelines [1]. Among these agents, tamsulosin is commonly prescribed because of its preferential affinity for the α1A-adrenergic receptor subtype and its relatively favorable cardiovascular safety profile [2,3]. Nevertheless, systemic vascular effects, including hypotension, may still occur.

Spinal anesthesia is commonly used for transurethral resection of the prostate (TUR-P), and the associated sympathetic blockade results in reductions in vascular tone that may be particularly relevant in older patients with limited cardiovascular compensatory reserve [4,5]. Previous studies have examined the perioperative hemodynamic effects of α1-blocker therapy in patients undergoing urological surgery under spinal anesthesia. Lee et al. prospectively compared patients receiving long-term tamsulosin with patients not receiving α1-blockers during TUR-P or HoLEP and evaluated serial blood pressure changes during dexmedetomidine sedation [6]. More recently, a prospective observational study evaluated hemodynamic responses in patients receiving selective or nonselective α1-blockers during urological surgery under spinal anesthesia, also in the context of dexmedetomidine administration [7]. These studies provide relevant evidence regarding α1-blocker use during spinal anesthesia; however, concomitant sedative administration may also influence hemodynamic responses, limiting the specific assessment of the association between chronic tamsulosin therapy and spinal anesthesia-related blood pressure changes.

Additional perioperative evidence has emerged from other anesthetic settings. A recent observational study in cardiac surgical patients reported greater vasoactive support among patients receiving preoperative tamsulosin [8]. However, the substantially different physiological and anesthetic conditions of cardiac surgery limit direct comparison with patients undergoing TUR-P under spinal anesthesia.

Thus, evidence specifically examining chronic tamsulosin therapy and hemodynamic responses during TUR-P under spinal anesthesia without concomitant sedative administration remains limited. Given that residual α1-adrenergic blockade may attenuate compensatory sympathetic vasoconstrictor responses to spinal anesthesia-induced reductions in vascular tone, we hypothesized that chronic tamsulosin therapy would be associated with greater reductions in arterial blood pressure following spinal anesthesia. Therefore, the present prospective observational study aimed to compare intraoperative hemodynamic changes between patients receiving chronic tamsulosin therapy and controls undergoing elective TUR-P under a standardized spinal anesthetic technique without intraoperative sedation.

## 2. Materials and Methods

### 2.1. Study Design

This single-center prospective observational study was conducted at a tertiary care hospital in İzmir, Türkiye, between June 2025 and January 2026. The study was approved by the Ethics Committee of Tepecik Training and Research Hospital (Decision No. 2023/01-10). Although ethical approval was obtained in 2023, patient enrollment was postponed due to logistical reasons. The study was subsequently registered at ClinicalTrials.gov (NCT06998537) before patient recruitment, and all participants were enrolled after registration. The final implemented study protocol differed from the initially registered protocol. The initial registration included a broader population of ASA I–III patients undergoing elective urologic surgery and exposure to different α1-adrenergic blockers. Before enrollment of the first study participant, the protocol was refined to establish a more standardized and clinically homogeneous study population. Accordingly, the implemented prospective study was restricted to ASA I–II patients undergoing TUR-P and to chronic tamsulosin exposure. The hemodynamic assessment protocol was also standardized to include MAP measurements at baseline and at 5, 15, 30, 45, and 60 min, with hypotension defined as MAP < 65 mmHg or a >20% decrease from baseline. These protocol refinements were made before enrollment of the first study participant, and data were subsequently collected according to this implemented protocol from the first enrolled participant onward. After enrollment had begun, data from the first 20 participants were used solely for the formal sample-size calculation, as described in Section 2.7. The prospective objective from the outset was to evaluate the between-group pattern of MAP changes following spinal anesthesia using these predefined measurement time points. However, the public registry record was not subsequently amended to reflect these changes. Written informed consent was obtained from all patients before surgery.

### 2.2. Patient Selection

Adult patients (≥18 years) with American Society of Anesthesiologists (ASA) physical status I–II who were scheduled for elective transurethral resection of the prostate (TUR-P) under spinal anesthesia were prospectively enrolled.

Patients were excluded if spinal anesthesia could not be performed, or if they had a history of ischemic heart disease, heart failure, clinically significant arrhythmias, neurological or psychiatric disorders, or any condition affecting the brain or spinal cord. Patients receiving regular antihypertensive medications or other vasoactive medications that could influence perioperative hemodynamic responses, other than chronic tamsulosin therapy, were also excluded. None of the patients in the tamsulosin group were receiving concomitant finasteride, dutasteride, or other BPH-specific medications.

Patients in the study group had been receiving chronic tamsulosin therapy for the management of lower urinary tract symptoms secondary to benign prostatic hyperplasia. All patients in Group 1 were receiving tamsulosin at a standard maintenance dose of 0.4 mg once daily, and the duration of tamsulosin therapy before surgery was recorded for each patient. Treatment adherence was assessed based on patient self-report, and patients reported regular daily use. Tamsulosin therapy was continued without temporary discontinuation before surgery. According to the institutional perioperative protocol, patients in Group 1 took their last dose at 22:00 on the evening before surgery, and no additional dose was administered on the morning of surgery.

### 2.3. Study Groups

Patients were classified into two groups according to preoperative chronic tamsulosin exposure:

Group 1: Patients receiving chronic tamsulosin therapy.

Group 2: Patients with no history of α1-adrenergic blocker use; for whom surgical management was planned as the initial treatment approach.

### 2.4. Perioperative Anesthesia Management

All patients underwent a standardized preoperative anesthetic assessment. Demographic characteristics including age, body mass index (BMI), and ASA physical status were recorded.

Standard intraoperative monitoring consisted of non-invasive blood pressure (NIBP), heart rate (HR), and peripheral oxygen saturation (SpO_2_). NIBP was measured at 5 min intervals as part of routine intraoperative monitoring.

Spinal anesthesia was performed at the L3–L4 interspace using a 25-gauge Quincke spinal needle with the patient in the sitting position. Hyperbaric 0.5% bupivacaine (10 mg) was administered intrathecally. Patients were subsequently positioned supine and received supplemental oxygen at 3 L/min via nasal cannula.

All patients received an isotonic crystalloid infusion at a maintenance rate of approximately 6 mL/kg/h according to the institutional protocol, without routine preoperative crystalloid preloading. When hypotension occurred, intravenous crystalloid boluses (typically 250 mL administered over 10 min) were administered according to clinical assessment by the attending anesthesiologist. If hypotension persisted following fluid administration on subsequent blood pressure measurement or clinical reassessment, intravenous ephedrine was administered in 5 mg boluses. Bradycardia was treated with intravenous atropine, administered in 0.5 mg doses according to clinical response. The same fluid management and treatment approach was applied to both study groups. Phenylephrine was not used in any patient.

The spinal anesthetic technique and local anesthetic dose were identical in both groups, targeting a sensory block level of approximately T10. The sensory block level was assessed bilaterally using pinprick testing before the start of surgery, and the highest achieved sensory level was recorded. No intraoperative sedative agents were administered to any patient during the study period.

For study analysis, systolic blood pressure (SBP), diastolic blood pressure (DBP), mean arterial pressure (MAP) and HR values were recorded at baseline before spinal anesthesia and at predefined time points of 5, 15, 30, 45, and 60 min after intrathecal injection.

### 2.5. Definitions

Hypotension was defined as either a reduction in MAP exceeding 20% from baseline or an absolute MAP below 65 mmHg. No predefined duration criterion was required for the definition of hypotension. Bradycardia was defined as HR < 50 beats/min.

### 2.6. Outcome Measures

#### 2.6.1. Primary Outcome

The primary outcome was the trajectory of mean arterial pressure (MAP) evaluated across predefined time points (5, 15, 30, 45, and 60 min) following spinal anesthesia, analyzed using a linear mixed-effects model incorporating group, time, and group × time interaction.

#### 2.6.2. Secondary Outcomes

Secondary outcomes included the incidence of intraoperative hypotension and bradycardia. Changes in systolic blood pressure (SBP) and diastolic blood pressure (DBP) across the predefined time points were evaluated as exploratory outcomes.

### 2.7. Statistical Analysis

Statistical analyses were performed using IBM SPSS Statistics version 21.0 (IBM Corp., Armonk, NY, USA). Categorical variables are presented as frequencies and percentages, whereas continuous variables are expressed as mean ± standard deviation (SD) or median (interquartile range [IQR]), depending on data distribution. Normality was assessed using the Kolmogorov–Smirnov test. There were no missing data for the variables included in the analysis.

Comparisons between the two groups were performed using the independent-samples *t* test for normally distributed continuous variables and the Mann–Whitney *U* test for non-normally distributed variables. Categorical variables were compared using Pearson’s chi-square test or Fisher’s exact test, as appropriate. A two-sided *p* value < 0.05 was considered statistically significant. To evaluate longitudinal changes in MAP while accounting for repeated measurements within individuals, a linear mixed-effects model for repeated measures was performed. Group, time, and the group × time interaction were included as fixed effects. Time was modeled as a categorical fixed factor representing the six predefined measurement time points (baseline, 5, 15, 30, 45, and 60 min). The model was adjusted for age, BMI, ASA physical status, and highest sensory block level. ASA physical status was entered as a categorical variable, with ASA I as the reference category. A first-order autoregressive [AR(1)] covariance structure was used to model within-subject correlations across repeated measurements, and denominator degrees of freedom were estimated using the Satterthwaite approximation. Adjusted estimated marginal means (EMMs) and between-group differences in MAP with 95% confidence intervals were derived from the linear mixed-effects model at each predefined time point. Serial SBP and DBP comparisons were considered exploratory analyses, and no multiplicity adjustment was applied to these exploratory comparisons.

At the initial stage of the study, data from the first 20 patients (10 patients per group) were used to perform a formal sample size calculation. Based on the observed between-group difference in MAP at 30 min, which corresponded to an effect size (Cohen’s d) of 0.54, the minimum required sample size was calculated using G*Power version 3.1.9.7 (Heinrich-Heine-Universität Düsseldorf, Düsseldorf, Germany). Using an independent-samples t-test with a two-sided α level of 0.05 and 80% statistical power, the analysis indicated that at least 108 patients (54 per group) were required. The sample size calculation was therefore based on a single representative between-group MAP comparison and was not specifically derived for the longitudinal mixed-effects model. The mixed-effects model was used for the primary analysis because it incorporated all repeated MAP measurements and accounted for within-subject correlation over time. This study was reported in accordance with the Strengthening the Reporting of Observational Studies in Epidemiology (STROBE) guidelines.

## 3. Results

**Figure 1:**
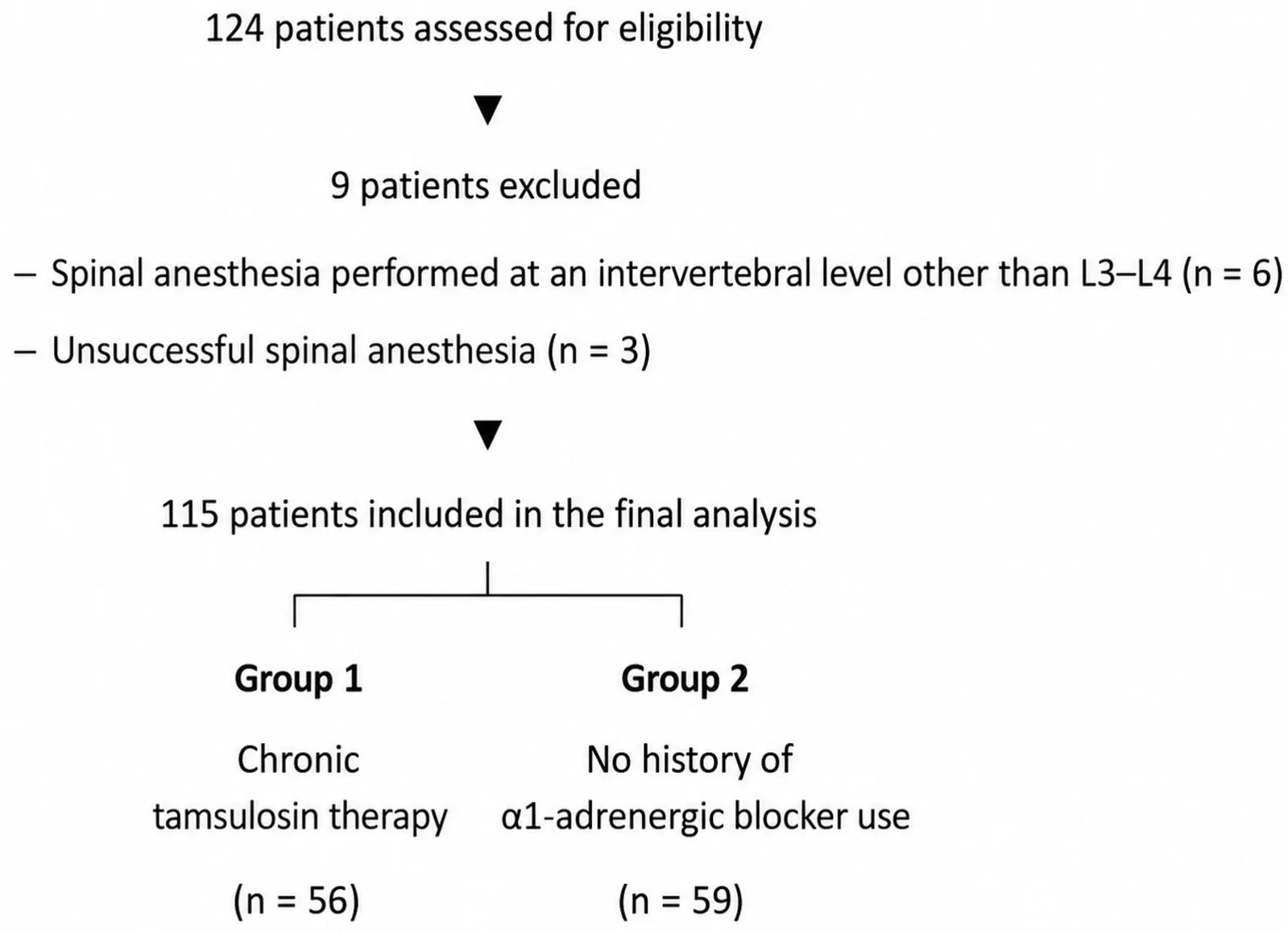
Flow diagram of patient inclusion and exclusion.

A total of 124 patients scheduled for elective transurethral resection of the prostate (TUR-P) under spinal anesthesia were screened for eligibility. Nine patients were excluded: six because spinal anesthesia was performed at an intervertebral level other than L3–L4 and three because of unsuccessful spinal anesthesia. Consequently, 115 patients were included in the final analysis. Of these, 56 patients receiving chronic tamsulosin therapy comprised the tamsulosin group (Group 1), while 59 with no history of α1-adrenergic blocker use comprised the control group (Group 2). The patient inclusion and exclusion process is shown in Figure 1.

The baseline demographic and clinical characteristics of the study groups are summarized in Table 1. The two groups were comparable regarding age, BMI, ASA physical status, and operative time. Patients in Group 1 had received tamsulosin at a maintenance dose of 0.4 mg once daily before surgery. The duration of chronic tamsulosin therapy ranged from 18 to 42 months, with a median of 29 months (IQR: 25–33). The mean interval between the last tamsulosin dose and the start of surgery was 13.5 ± 1.5 h. The two groups were also comparable with respect to the highest sensory block level achieved after spinal anesthesia. The total volume of intraoperative crystalloid administered was also comparable between Group 1 and Group 2 (Table 1).

Hypotension occurred in 21 of 56 patients (37.5%) in the tamsulosin group and in 17 of 59 patients (28.8%) in the control group, with no statistically significant difference between the groups (*p* = 0.32). Ephedrine was required in 5 patients (8.9%) in Group 1 and 3 patients (5.1%) in Group 2, with no statistically significant difference between the groups (*p* = 0.48; Table 1). Among patients requiring rescue ephedrine, in Group 1 (*n* = 5), three patients received two 5 mg boluses (cumulative dose, 10 mg per patient), and two patients received a single 5 mg bolus, resulting in a total of eight boluses (cumulative dose, 40 mg). In Group 2 (*n* = 3), one patient received two 5 mg boluses (cumulative dose, 10 mg), and two patients received a single 5 mg bolus, resulting in a total of four boluses (cumulative dose, 20 mg). Bradycardia occurred in 3 patients (5.4%) in Group 1 and 1 patient (1.7%) in Group 2 (*p* = 0.36; Table 1). Regarding treatment for these episodes, in Group 1, two patients received a single 0.5 mg dose of intravenous atropine and one patient received two 0.5 mg doses, resulting in a total of four doses (cumulative dose, 2.0 mg) administered to three patients. In Group 2, the single affected patient received a single 0.5 mg dose of atropine (cumulative dose, 0.5 mg).

Heart rate values remained comparable between the two groups throughout the study period. No statistically significant differences were observed at baseline or at 5, 15, 30, 45, and 60 min after spinal anesthesia (all *p* > 0.05) (Table 2). Bradycardia developed in three patients (5.4%) in Group 1 and one patient (1.7%) in Group 2, with no significant difference between the groups (*p* = 0.36).

**Figure 2:**
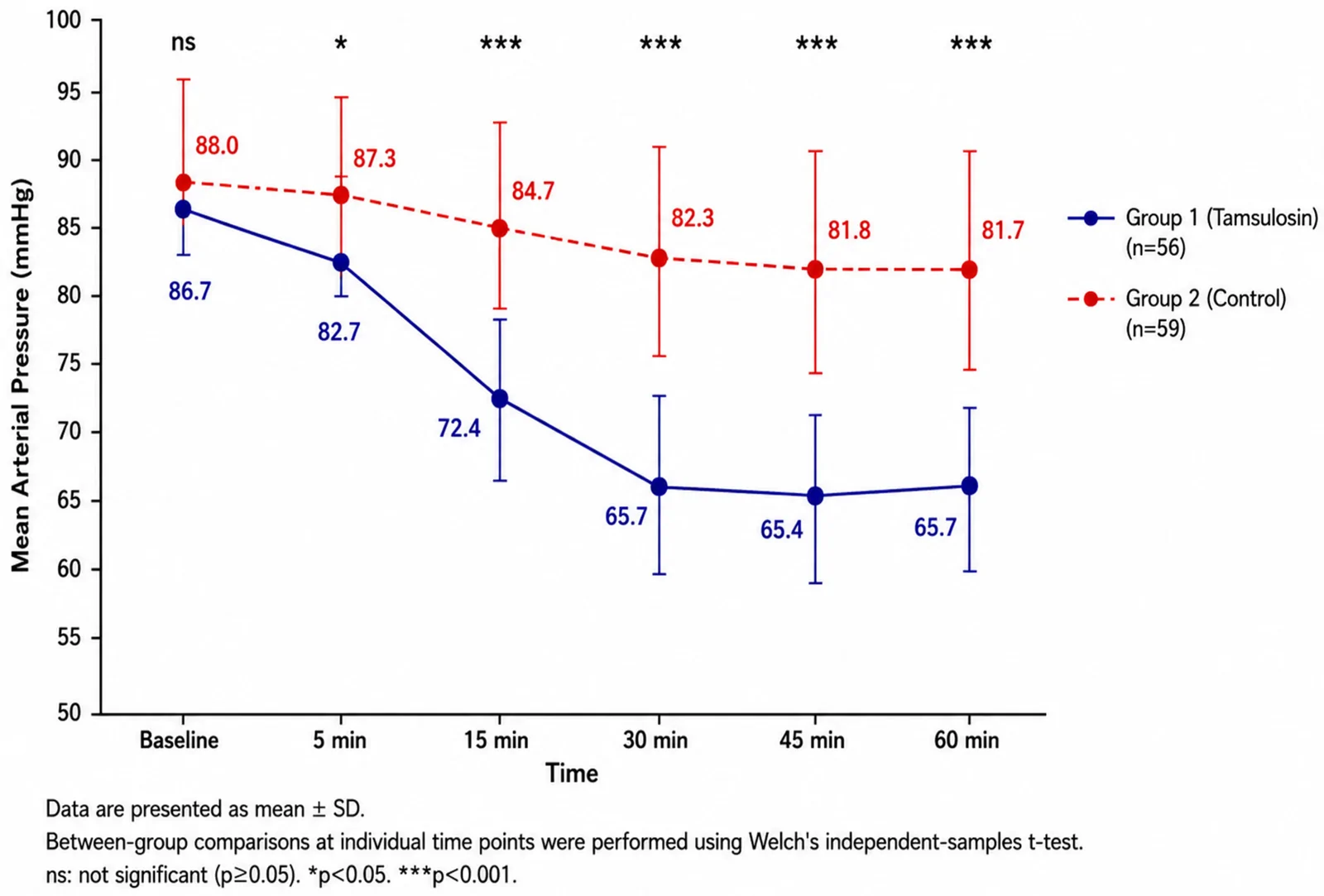
Comparison of mean arterial pressure (MAP) over time.

Model-derived adjusted estimated marginal means (EMMs) and between-group differences in MAP at each predefined time point are presented in Table 3. Baseline adjusted MAP was comparable between the groups (adjusted mean difference [Group 1 − Group 2], −1.5 mmHg; 95% CI −5.3 to 2.3; *p* = 0.432). Following spinal anesthesia, the adjusted between-group difference increased over time, reaching −4.9 mmHg (95% CI −8.8 to −1.0; *p* = 0.015) at 5 min, −12.7 mmHg (95% CI −16.2 to −9.2; *p* < 0.001) at 15 min, −17.0 mmHg (95% CI −20.2 to −13.8; *p* < 0.001) at 30 min, −16.8 mmHg (95% CI −19.9 to −13.7; *p* < 0.001) at 45 min, and −16.4 mmHg (95% CI −19.5 to −13.3; *p* < 0.001) at 60 min (Table 3). The adjusted linear mixed-effects model demonstrated a significant overall group × time interaction for MAP (F(5, 565.10) = 8.34, *p* < 0.001), indicating that MAP trajectories differed between the groups over time (Figure 2). In the adjusted linear mixed-effects model, time was treated as a categorical fixed factor, with baseline as the reference time point. In the control group, MAP decreased relative to baseline from 15 min onward (15 min: B = −3.20, 95% CI −4.26 to −2.14; 30 min: B = −5.60, 95% CI −6.68 to −4.52; 45 min: B = −6.10, 95% CI −7.18 to −5.02; and 60 min: B = −6.20, 95% CI −7.30 to −5.10; all *p* < 0.001), whereas the 5 min change was not statistically significant (B = −0.70, 95% CI −1.72 to 0.32; *p* = 0.178). The negative Group × Time interaction coefficients indicated greater reductions in MAP from baseline in the tamsulosin group relative to the control group at each intraoperative measurement time point (5 min: B = −3.40; 15 min: B = −11.20; 30 min: B = −15.50; 45 min: B = −15.30; and 60 min: B = −14.90; all *p* < 0.001) (Table 4). Age, BMI, ASA physical status, and highest sensory block level were not statistically significant covariates.

A sensitivity analysis was performed after excluding the eight patients who received rescue ephedrine (five in the tamsulosin group and three in the control group; *n* = 107). The adjusted linear mixed-effects model demonstrated a significant overall group × time interaction for MAP (F(5, 523.40) = 8.12, *p* < 0.001). The magnitude and direction of the adjusted between-group MAP differences remained similar to those observed in the primary analysis (Table 5).

## 4. Discussion

The present prospective observational study evaluated the association between chronic tamsulosin therapy and intraoperative hemodynamic changes in patients undergoing elective transurethral resection of the prostate under spinal anesthesia. The principal finding was that patients receiving chronic tamsulosin therapy exhibited a lower MAP profile during the first 60 min following spinal anesthesia, with adjusted between-group differences in MAP emerging after spinal anesthesia, increasing up to 30 min, and remaining substantial thereafter. Notably, the incidence of criteria-defined hypotension was higher than the actual requirement for rescue ephedrine. A distinction between the incidence of hypotension and rescue ephedrine requirement has also been reported by Biricik et al.; hypotension occurred in 70% and 67.5% of patients in the norepinephrine and phenylephrine groups, respectively, whereas rescue ephedrine was required in 42.5% and 22.5% [9]. Although their treatment protocol and study population differed from ours, these findings illustrate that the incidence of recorded hypotension and the requirement for rescue vasopressor therapy may represent distinct clinical outcomes. In the present study, rescue ephedrine was administered when hypotension persisted following fluid administration and clinical reassessment. This approach is consistent with the broader clinical management of spinal anesthesia-induced hypotension, which may include fluid administration and vasopressor therapy according to the clinical context [10]. These findings suggest an association between chronic tamsulosin therapy and lower arterial pressure during the observation period following spinal anesthesia. Although clinically defined hypotension was numerically more frequent in the tamsulosin group, the between-group difference was not statistically significant, and rescue ephedrine use remained limited in both groups. Importantly, the incidence of criteria-defined hypotension represents a patient-level binary outcome and should be distinguished from the group-level mean MAP values observed at individual measurement time points. Furthermore, the sensitivity analysis excluding patients who received rescue ephedrine yielded findings consistent with the primary analysis, supporting the robustness of the observed between-group differences in MAP trajectories. The approximately 17-mmHg adjusted between-group difference in MAP observed at 30–45 min is clinically notable, particularly as the adjusted mean MAP in the tamsulosin group approached the commonly used hypotension threshold of 65 mmHg during this period. However, this hemodynamic difference was not accompanied by a statistically significant increase in the incidence of criteria-defined hypotension or rescue vasopressor requirements. Furthermore, because our study was not primarily designed or powered to evaluate postoperative organ injury or other patient-centered outcomes, this observed MAP difference should be interpreted as a distinct hemodynamic response rather than direct evidence of clinically important harm. Future prospective investigations incorporating broader postoperative clinical endpoints are warranted to clarify the clinical implications of this finding.

Hypotension is a well-recognized consequence of spinal anesthesia and is mainly attributed to sympathetic blockade, resulting in arterial and venous vasodilation with subsequent reductions in systemic vascular resistance [4,5]. These physiological changes may be more pronounced in older patients because age-related alterations in autonomic regulation and cardiovascular reserve may limit compensatory responses to neuraxial blockade [11,12]. Recent prospective evidence has further demonstrated that spinal anesthesia-induced hypotension remains particularly relevant in older surgical patients, supporting the importance of age-related susceptibility to hemodynamic instability after neuraxial blockade [13].

Tamsulosin is among the most frequently prescribed α1-adrenergic receptor antagonists for the treatment of benign prostatic hyperplasia because of its favorable efficacy and safety profile [2,3,14]. Owing to its preferential affinity for the α1A receptor subtype, tamsulosin is generally considered to have fewer vascular effects than non-selective α1-blockers [2,14]. Nevertheless, α1A selectivity may not completely eliminate systemic hemodynamic effects under all clinical circumstances. Although no additional dose was administered on the morning of surgery, tamsulosin has a relatively long apparent half-life of approximately 14–15 h; therefore, pharmacologically relevant α1-adrenergic receptor blockade may have persisted during the intraoperative period after the last dose taken on the evening before surgery [15]. During spinal anesthesia, reduced sympathetic tone together with pharmacological α1-adrenergic receptor blockade could potentially attenuate compensatory vasoconstrictive responses, which may partially explain the lower intraoperative arterial pressure values observed in the present study. Our findings complement previous prospective studies evaluating α1-blockers during spinal anesthesia [6,7]. However, these studies incorporated dexmedetomidine sedation, which may independently influence hemodynamic responses. In contrast, no intraoperative sedation was used in the present study, reducing this potential confounding influence. Recent observational evidence from cardiac surgery has also demonstrated an association between preoperative tamsulosin use and increased intraoperative vasoactive support [8], although the markedly different anesthetic and physiological setting limits direct comparison with the present findings. In addition, the highest sensory block levels achieved after spinal anesthesia were comparable between the two groups. Therefore, differences in block height are unlikely to fully account for the observed differences in intraoperative blood pressure, although their potential contribution cannot be completely excluded.

Although tamsulosin is generally considered a uroselective α1A-adrenergic receptor antagonist with a relatively favorable cardiovascular safety profile, perioperative hypotension has been described in several clinical reports. Bird et al. demonstrated an increased risk of severe hypotension, particularly during the early period following initiation of tamsulosin therapy, suggesting that systemic vascular effects may still occur despite receptor selectivity [16]. Similarly, Lee et al. reported life-threatening hypotension in a patient with spinal cord injury receiving tamsulosin, with blood pressure returning to normal after discontinuation of the drug [17]. These observations are broadly consistent with the lower intraoperative arterial pressure values observed in the present study, although direct comparisons should be made cautiously because of differences in patient populations and clinical settings.

Previous reports have also described perioperative hypotension associated with tamsulosin under general anesthesia. Kumar et al. described severe hypotension immediately after induction of general anesthesia in a patient receiving chronic tamsulosin therapy for benign prostatic hyperplasia, requiring repeated administration of ephedrine throughout the procedure [18]. Similarly, Chauhan et al. reported profound intraoperative hypotension in a patient receiving long-term high-dose tamsulosin during general anesthesia [19]. In both cases, the interaction between anesthetic-induced vasodilation and the pharmacological effects of tamsulosin was considered a possible explanation for the observed hemodynamic instability. Although the anesthetic technique differed from that used in the present study, these reports support the possibility that chronic tamsulosin therapy may influence perioperative blood pressure responses under different anesthetic conditions.

The present findings should also be interpreted in the context of the overall vascular safety profile of α1-adrenergic receptor antagonists. In a meta-analysis, Nickel et al. reported a higher incidence of vascular adverse events, including dizziness, hypotension, and syncope, among patients receiving α1-blockers compared with placebo, although the magnitude of these effects differed among individual agents [20]. While tamsulosin is generally regarded as having a more favorable cardiovascular safety profile than non-selective α1-blockers, the present findings indicate that chronic tamsulosin use may be associated with lower intraoperative arterial pressure during spinal anesthesia.

Hartmann et al. identified advanced age as one of the major determinants of hypotension following spinal anesthesia [11]. In the present study, the median age of the study groups was approximately 65 years, suggesting that age-related physiological changes may have increased the overall susceptibility to intraoperative hypotension. However, because the age distribution and sensory block levels were comparable between the groups, these factors are less likely to fully explain the lower intraoperative arterial pressure values observed in patients receiving chronic tamsulosin therapy.

One of the strengths of the present study is that only patients receiving chronic tamsulosin therapy at a standardized maintenance dose of 0.4 mg once daily were included. Furthermore, the timing of the last preoperative dose was standardized according to the institutional perioperative protocol. These methodological features helped minimize pharmacological heterogeneity and allowed a more focused evaluation of the association between chronic tamsulosin therapy and intraoperative hemodynamic changes.

### Study Limitations

The present study has several limitations. First, although the prospective observational design allowed standardized perioperative data collection, it does not permit causal inferences regarding the relationship between chronic tamsulosin therapy and intraoperative hemodynamic changes. Second, detailed information on potentially relevant comorbid conditions, including hypertension, diabetes mellitus, coronary artery disease, and chronic kidney disease, as well as hemoglobin concentration and laboratory-based renal function parameters, was not systematically collected as prespecified study variables. Therefore, these factors could not be incorporated into the adjusted analyses, and residual confounding related to unmeasured clinical characteristics cannot be excluded. Previous exposure to other α1-adrenergic blockers was not systematically recorded and therefore could not be evaluated. In addition, restriction of the study population to ASA physical status I–II patients and exclusion of patients receiving regular antihypertensive or other vasoactive medications resulted in a selected study population and may limit the generalizability of the findings to the broader TUR-P population. TUR-P-specific perioperative variables, including irrigation fluid volume, resection weight, estimated blood loss, and perioperative hemoglobin changes, were also not systematically collected and therefore could not be evaluated as potential contributors to hemodynamic changes. Third, because the sample size was powered only for the primary MAP outcome, the study may have had limited statistical power for secondary outcomes, including hypotension. Consequently, the absence of a significant difference should not be interpreted as evidence of equivalence. Hemodynamic outcomes were analyzed during the predefined 60 min observation period following spinal anesthesia. Accordingly, the findings primarily reflect the hemodynamic response during this early period following spinal anesthesia. Finally, the single-center design may limit the generalizability of the findings.

## 5. Conclusions

In this prospective observational study, lower mean arterial pressure values were observed during the observation period following spinal anesthesia in patients receiving chronic tamsulosin therapy who underwent transurethral resection of the prostate. Despite the differences observed in continuous blood pressure measurements, the incidences of clinically defined hypotension and bradycardia did not differ significantly between the groups. These findings suggest that patients receiving chronic tamsulosin therapy may experience lower arterial pressure following spinal anesthesia. However, given the observational design of the study, these findings should be interpreted with caution. Larger multicenter prospective studies are needed to confirm these findings and determine their clinical implications.

## Figures and Tables

**Table 1:** Demographic and clinical characteristics of the study groups.

	Group 1 (*n* = 56)	Group 2 (*n* = 59)	*p* Value
Age (years, median) (IQR)	64 (58–71.25)	66 (58–71.5)	0.86
ASA physical status, *n* (%)			0.68
I	21 (37.50%)	20(33.89%)	
II	35 (62.50%)	39 (66.10%)	
Smoking status, *n* (%)			0.81
Yes	32 (57.1%)	35 (59.3%)	
No	24 (42.9%)	24 (40.7%)	
Body mass index (kg/m^2^, mean ± SD)	24.62 ± 3.32	24.94 ± 3.37	0.61
Baseline SBP (mmHg), mean ± SD	120.9 ± 12.8	122.5 ± 13.5	0.51
Baseline DBP (mmHg), mean ± SD	69.6 ± 8.4	70.8 ± 9.2	0.46
Baseline MAP (mmHg), mean ± SD	86.7 ± 9.9	88.0 ± 11.4	0.50
Baseline HR (beats/min), median (IQR)	72 (66–80)	73 (65–81)	1.00
Highest sensory block level, median (IQR)	T9 (T8–T10)	T10 (T9–T11)	0.51
Duration of tamsulosin therapy, months, median (IQR)	29 (25–33)	-	
Operative time (min), mean ± SD	113.57 ± 15.07	113.17 ± 13.18	0.87
Intraoperative crystalloid volume (mL)	2180.00 ± 727	2086.67 ± 487	0.42
Hypotension, *n* (%)	21 (37.5%)	17 (28.8%)	0.32
Ephedrine requirement, *n* (%)	5 (8.9%)	3 (5.1%)	0.48
Bradycardia, *n* (%)	3 (5.4%)	1 (1.7%)	0.36

Data are presented as mean ± SD, median (IQR), or *n* (%), as appropriate. – indicates not applicable. BMI: body mass index; ASA: American Society of Anesthesiologists; SBP: systolic blood pressure; DBP: diastolic blood pressure; MAP: mean arterial pressure; HR: heart rate. Group 1: chronic tamsulosin therapy; Group 2: control (no α1-adrenergic blocker use).

**Table 2:** Comparison of heart rate values between the study groups.

Heart Rate (beats/min), Median (IQR)	Group 1 (*n* = 56)	Group 2 (*n* = 59)	*p* Value
Baseline	72 (66–80)	73 (65–81)	1.000
5 min	70 (65–76)	72 (64–80)	0.368
15 min	69 (63–75)	72 (65–85)	0.490
30 min	68 (63–76)	71 (64–80)	0.211
45 min	67 (63–75)	71 (64–80)	0.153
60 min	68 (64–75)	69 (63–76)	0.981

Data are presented as median (IQR). HR: heart rate. Group 1: chronic tamsulosin therapy; Group 2: control (no α1-adrenergic blocker use).

**Table 3:** Adjusted estimated marginal means and between-group differences in mean arterial pressure (MAP) over time derived from the linear mixed-effects model.

Time Point	Group 1 (Tamsulosin), Adjusted EMM (95% CI)	Group 2 (Control), Adjusted EMM (95% CI)	Adjusted Mean Difference (95% CI), mmHg	*p* Value
Baseline	86.6 (84.0–89.2)	88.1 (85.6–90.6)	−1.5 (−5.3 to 2.3)	0.432
5 min	82.5 (79.9–85.1)	87.4 (84.9–89.9)	−4.9 (−8.8 to −1.0)	0.015
15 min	72.2 (69.6–74.8)	84.9 (82.4–87.4)	−12.7 (−16.2 to −9.2)	<0.001
30 min	65.5 (62.9–68.1)	82.5 (80.0–85.0)	−17.0 (−20.2 to −13.8)	<0.001
45 min	65.2 (62.6–67.8)	82.0 (79.5–84.5)	−16.8 (−19.9 to −13.7)	<0.001
60 min	65.5 (62.9–68.1)	81.9 (79.4–84.4)	−16.4 (−19.5 to −13.3)	<0.001

Data are presented as adjusted estimated marginal means (EMMs) with 95% confidence intervals (CIs), derived from the linear mixed-effects model adjusted for age, BMI, ASA physical status, and highest sensory block level. Group 1: chronic tamsulosin therapy; Group 2: control (no α1-adrenergic blocker use). Adjusted mean differences are presented as Group 1 minus Group 2 with 95% CIs. EMM: estimated marginal mean; MAP: mean arterial pressure; CI: confidence interval.

**Table 4:** Fixed-effect parameter estimates from the linear mixed-effects model for mean arterial pressure.

Fixed Effect/Parameter	B	SE	t	*p* Value	95% CI
Intercept	88.10	1.25	70.48	<0.001	85.65 to 90.55
Group (Tamsulosin vs Control)	−1.50	1.95	−0.77	0.432	−5.32 to 2.32
Time (reference: Baseline)					
5 min	−0.70	0.52	−1.35	0.178	−1.72 to 0.32
15 min	−3.20	0.54	−5.93	<0.001	−4.26 to −2.14
30 min	−5.60	0.55	−10.18	<0.001	−6.68 to −4.52
45 min	−6.10	0.55	−11.09	<0.001	−7.18 to −5.02
60 min	−6.20	0.56	−11.07	<0.001	−7.30 to −5.10
Group × Time Interaction					
Tamsulosin × 5 min	−3.40	0.74	−4.59	<0.001	−4.85 to −1.95
Tamsulosin × 15 min	−11.20	0.76	−14.74	<0.001	−12.69 to −9.71
Tamsulosin × 30 min	−15.50	0.77	−20.13	<0.001	−17.01 to −13.99
Tamsulosin × 45 min	−15.30	0.77	−19.87	<0.001	−16.81 to −13.79
Tamsulosin × 60 min	−14.90	0.78	−19.10	<0.001	−16.43 to −13.37
Age	0.18	0.14	1.29	0.198	−0.09 to 0.45
BMI	−0.04	0.03	−1.33	0.184	−0.10 to 0.02
ASA Physical Status (ASA II vs. ASA I)	0.09	0.11	0.82	0.413	−0.13 to 0.31
Highest Sensory Block Level	0.03	0.06	0.50	0.617	−0.09 to 0.15

Note: B, unstandardized coefficient; SE, standard error; CI, confidence interval; BMI, body mass index; ASA, American Society of Anesthesiologists. Time was modeled as a categorical fixed factor, with baseline as the reference time point. The control group served as the reference group for the group effect. Time coefficients represent changes from baseline in the control group, whereas Group × Time interaction coefficients represent the additional change from baseline in the tamsulosin group relative to the control group at each corresponding time point. The model was adjusted for age, BMI, ASA physical status, and highest sensory block level. ASA physical status was modeled as a categorical variable, with ASA I as the reference category. The omnibus test for the overall group × time interaction was statistically significant (F(5, 565.10) = 8.34, *p* < 0.001).

**Table 5:** Sensitivity analysis of mean arterial pressure (MAP) trajectories excluding patients who received rescue ephedrine (*n* = 107).

Time Point	Group 1 (Tamsulosin, *n* = 51) Adjusted EMM (95% CI)	Group 2 (Control, *n* = 56) Adjusted EMM (95% CI)	Adjusted Mean Difference (95% CI), mmHg	*p* Value
Baseline	86.2 (83.5 to 88.9)	87.7 (85.1 to 90.3)	−1.5 (−5.2 to 2.2)	0.428
5 min	81.8 (79.1 to 84.5)	87.1 (84.5 to 89.7)	−5.3 (−9.1 to −1.5)	0.007
15 min	71.1 (68.4 to 73.8)	84.2 (81.6 to 86.8)	−13.1 (−16.8 to −9.4)	<0.001
30 min	64.2 (61.5 to 66.9)	81.9 (79.3 to 84.5)	−17.7 (−21.1 to −14.3)	<0.001
45 min	63.9 (61.2 to 66.6)	81.2 (78.6 to 83.8)	−17.3 (−20.7 to −13.9)	<0.001
60 min	64.4 (61.7 to 67.1)	81.0 (78.4 to 83.6)	−16.6 (−19.9 to −13.3)	<0.001

Note: Data are presented as adjusted estimated marginal means (EMMs) with 95% confidence intervals (CIs), derived from the linear mixed-effects model adjusted for age, BMI, ASA physical status, and highest sensory block level. A sensitivity analysis excluding patients who received rescue ephedrine (Group 1, *n* = 5; Group 2, *n* = 3) was performed to assess the robustness of the primary findings to rescue vasopressor administration. The overall group × time interaction remained statistically significant (F(5, 523.40) = 8.12, *p* < 0.001).

## Data Availability

The datasets generated and/or analyzed during the current study are available from the corresponding author upon reasonable request.
